# The use of empirical research in bioethics: a survey of researchers in twelve European countries

**DOI:** 10.1186/s12910-017-0239-0

**Published:** 2017-12-22

**Authors:** Tenzin Wangmo, Veerle Provoost

**Affiliations:** 1 Institute for Biomedical Ethics, University of Basel, Basel, Switzerland; 2 Bioethics Institute Ghent, University of Ghent, Ghent, Belgium

**Keywords:** Empirical bioethics, Empirical research, Bioethics, Qualitative research, Quantitative research

## Abstract

**Background:**

The use of empirical research methods in bioethics has been increasing in the last decades. It has resulted in discussions about the ‘empirical turn of bioethics’ and raised questions related to the value of empirical work for this field, methodological questions about its quality and rigor, and how this integration of the normative and the empirical can be achieved. The aim of this paper is to describe the attitudes of bioethics researchers in this field towards the use of empirical research, and examine their actual conduct: whether they use empirical research methods (and if so, what methods), and whether (and how) they have made attempts at integrating the empirical and the normative.

**Methods:**

An anonymous online survey was conducted to reach scholars working in bioethics/biomedical ethics/ethics institutes or centers in 12 European countries. A total of 225 bioethics researchers participated in the study. Of those, 200 questionnaires were fully completed, representing a response rate of 42.6%. The results were analysed using descriptive statistics.

**Results:**

Most respondents (*n* = 175; 87.5%) indicated that they use or have used empirical methods in their work. A similar proportion of respondents (61.0% and 59.0%) reported having had at least some training in qualitative or quantitative methods, respectively. Among the ‘empirical researchers’, more than a fifth (22.9%) had not received any methodological training. It appears that only 6% or less of the ‘empirical researchers’ considered themselves experts in the methods (qualitative or quantitative) that they have used. Only 35% of the scholars who have used empirical methods reported having integrated empirical data with normative analysis, whereas for their current projects, 59.8% plan to do so.

**Conclusions:**

There is a need to evaluate the current educational programs in bioethics and to implement rigorous training in empirical research methods to ensure that ‘empirical researchers’ have the necessary skills to conduct their empirical research in bioethics. Also imperative is clear guidance on the integration of the normative and the empirical so that researchers who plan to do so have necessary tools and competences to fulfil their goals.

**Electronic supplementary material:**

The online version of this article (10.1186/s12910-017-0239-0) contains supplementary material, which is available to authorized users.

## Background

The use of empirical research in bioethics, broadly defined as the use of qualitative and/or quantitative research methods from the social sciences, has increased in the last few decades. Sugarman and colleagues [1] reported that 8% of the papers published between 1980 and 1984 used empirical methods and the proportion of empirical biomedical ethics publications in bioethics has increased to 16% in 2000–2005. Another study that captured the prevalence of empirical research in nine biomedical ethics journals concluded that the use of empirical research had increased from 5.4% in 1990 to 15.3% in 2003 [2]. This trend of increasing empirical work in bioethics is also evident with the emergence of journals devoted to publishing empirical studies in bioethics, such as AJOB Empirical Bioethics and Narrative Inquiry in Bioethics: A Journal of Qualitative Research. Newer journals like the Journal of Bioethical Inquiry (since 2004), BMC Medical Ethics (since 2000), and Developing World Bioethics (since 2001) are also embracing empirical work carried out in this field.

The empirical trend in bioethics has resulted in (at least) two kinds of debates: (a) whether the empirical trend is necessary and valuable; and (b) how the empirical data and normative ethics could (or should) be integrated. Several scholars have discussed how empirical findings can be combined with ethical norms [3–10]. Davies and colleagues [11] noted 32 distinct methodologies of empirical ethics highlighting the many ways of integrating the normative and the empirical. Such integration raises many questions since among other things, the diverse normative ethics viewpoints may result in different interpretations of the same empirical results [12, 13].

Although the value of empirical studies to the field of medical and clinical ethics have been highlighted [14, 15], doubts are raised about the quality of bioethics studies for which empirical research methods are used [16–18]. Specifically, scholars have underlined that these normative empirical integration must comply with two quality standards: the standards of a normative conceptual analysis and the epistemological standards of the parent discipline whose empirical methodology was used [16, 19].

In light of the lack of data about who uses empirical research methods in bioethics (and what methods are used) as well as the lack of clarity as to how the empirical and the normative is or can best be merged (due to the availability of several methods and their novel characteristics) [5–7, 20–23], we designed this study. Its aims are twofold: 1) to explore the attitudes of researchers in bioethics towards the use empirical research, and 2) to examine their actual empirical research conduct: whether they use empirical methods, if so, what methods they employ and what, if any, training they have had; and whether as well as how they have attempted to integrate the normative and the empirical. This study is thus a first attempt to provide a description of bioethics scholars’ empirical research conduct, the methods that they use, how they assess their methodological competencies, as well as their views about the use of empirical research in this field.

## Methods

### Selection of study participants

Using our network of experts and scholars working in the field of biomedical ethics and bioethics on the continent, we compiled a comprehensive list of 38 European bioethics institutes^1^ from 13 countries. We amended our list by adding members of the European Association of Centres of Medical Ethics *(*EACME) and the European Society for Philosophy of Medicine and Healthcare (ESPMH). Twenty-five institutes were added from 12 countries using the EACME list (7 countries were not previously represented), and one institute was added using the ESPMH list. In total, we identified 64 relevant bioethics institutes across 20 European countries. For each of the institutes on our list, we searched for the email addresses of their members. We included all countries for which contact information of members could be obtained in more than half of the centers identified in the particular country. This resulted in the inclusion of 12 countries: Belgium, Denmark, Germany, Ireland, Moldova, The Netherlands, Norway, Romania, Switzerland, Spain, Sweden, and United Kingdom. The study’s individual participants included all researchers working in bioethics institutes (listed on the institutes’ official websites or obtained via our expert networks) in the 12 countries selected above. Additional file 1 summarizes our sampling method.

### Materials

We constructed a questionnaire including three thematic sections: (a) respondents’ attitudes and views about empirical research in bioethics; (b) their use of empirical methods in bioethics and if never used, willingness/intention to use empirical methods in the future, and (c) general questions related to the (types of) empirical methods used in the past, specific questions related to the (types of) empirical methods planned for a current project, as well as the training in empirical research methods the researcher had. The last part also included questions about the integration of the empirical and the normative to find out if (and how) the respondents have made attempts to do so. The questionnaire concluded with a socio-demographic section. Survey logic was applied to automatically filter questions based on the participant responses to ensure all questions were relevant. The survey completion time was expected to be between 10 and 20 min. No incentives were provided for participation. For more details, we refer the readers to Additional file 2, the survey in its entirety along with the survey logic.

The survey and the cover letter were pilot tested with 10 researchers located in the EU and North America who were not part of the targeted sample of the survey. They ranged from recent graduates to experts in the field to mimic the characteristics of the study participants. The goal of the piloting was to test both the content (including wording of the questions) and the structure of the questionnaire (including efficiency of the layout). The responses of the piloting were positive. Only small changes were made to the wording of a few questions and one open-ended question was added.

### Data collection

A request to participate in the survey was sent via personalized email to all researchers listed as a faculty member, post-doc, research assistant, or PhD student on the websites of the bioethics institutes in the 12 countries. A total of 469 scholars from 35 bioethics institutes received a link to the online survey (using Qualtrics) during the first week of January 2017. The email introduced each potential participant to the study, explaining briefly that they were identified as eligible participants, informing them about the researchers, the study’s significance, and its anonymous nature. A reminder to complete the survey was sent during the third week of January 2017, followed by a final reminder at the end of January 2017. A final note of appreciation and conclusion of the survey was sent to all potential participants in February 2017.

### Response rate

A total of 225 participants took part in the survey. This accounts for an actual response rate of 47.9%. Incomplete surveys (25) were excluded, leaving 200 fully completed questionnaires for analysis. The final response rate for the study was 42.6%.

### Data analysis

Respondents’ data recorded through Qualtrics was exported to a data file compatible with Statistical Package of the Social Sciences, IBM SPSS.22. Responses to Likert-type scales (totally agree, agree, neutral, disagree, totally disagree) and similar variables were combined into fewer categories to facilitate the analysis. Descriptive analysis was conducted. We used Chi^2^ to compare the distribution of categorical variables among different groups of respondents (i.e. empirical researchers who had made an attempt at integrating the empirical and the normative with those who did not).

## Results

### Respondent characteristics

The 200 bioethicists who fully completed the questionnaire were from the 12 countries sampled in the study. The country response rate ranged from 14% (for Denmark) to 82% (for Moldova). Demographic characteristics of the study sample are presented in Table 1. The three most important educational disciplines of the respondents were philosophy (*n* = 59; 29.5%); bioethics or biomedical ethics (*n* = 33; 16.5%); and a combination of disciplines (e.g. philosophy and psychology; sociology and public health) (*n* = 32; 16%). We sought to compare the study sample (response group) with data on the non-response group for the variable that was sufficiently available for both groups (Table 1). The data for the latter group were collected via online biographical information.

**Table 1:** Demographic characteristics of the study sample and population

	Study sample (*N* = 200)		Study population (*N* = 469)^b^	
	*n*	%	*n*	%
Gender				
Man	83	40.5	198	45.0
Woman	117	58.5	242	55.0
Age category ^a^				
≤ 34 years	69	34.8	–	–
35–44 years	78	39.4	–	–
≥ 45 years	51	25.8	–	–
Education				
Master’s degree	39	19.5	–	–
PhD or equivalent	148	74.0	–	–
Other	13	6.5	–	–
Position				
Professor	57	28.5	–	–
Post-doc or senior researcher	81	40.5	–	–
PhD-students	62	31.0	–	–

^a^Data missing for two cases for ‘age category’ in the study sample

^b^Data for the population (all bioethics scholars who received our survey) contained missing values up to 70% (e.g. age) because this data was either not available on the public domain (institution websites) or provided using labels that did not allow categorisation (such as for data on background or degrees). Moreover, the data on education and position were mutually contradictory indicating that information online was not updated. We therefore could only use the data on gender (data missing for 29 cases for the study population) to compare the response group with the non-response group

Chi^2^ test was run to compare the study sample with the nonresponse (study population minus the study sample) for gender. No significant difference was found

### Methodological education and training

All respondents provided information on their methodological education, type of empirical work they have carried out, and years of experience with empirical work (see Table 2). We find that a similar proportion (61.0% and 59.0%) reported having taken at least a qualitative or quantitative methods course as part of their education, respectively. Approximately half of the respondents in the sample (*n* = 94; 47%) studied both qualitative and quantitative methods as part of their education, whereas comparable proportions of respondents studied either qualitative methods (28; 14%) or quantitative methods (24; 12%). A quarter of the scholars (*n* = 54; 27%) had no formal empirical methods training.

**Table 2:** Education in and experience with empirical research (*N* = 200)

	*n*	%
Qualitative methods as part of the education		
Yes	122	61.0
No	78	39.0
Quantitative methods as part of the education		
Yes	118	59.0
No	82	41.0
Type of methodological education^a^		
Only qualitative	28	14.0
Only quantitation	24	12.0
Mixed method	94	47.0
No methods education	54	27.0
Past experience in empirical work		
Ever collected empirical data	143	71.5
Ever analysed empirical data	149	74.5
Ever supervised empirical research	103	51.5
Years of experience with empirical research		
3 years or less	77	38.5
4–7 years	56	28.0
8 years or more	67	33.5
Experience as supervisor of PhD-students		
Yes	85	42.5
No	115	57.5
Proportion of work time spent on empirical work^b c^		
30% or less	116	58.9
31–60%	62	31.5
More than 60%	19	9.6
Proportion of work time spent on normative work^b c^		
30% or less	95	47.7
31–60%	57	28.6
More than 60%	47	23.6
Willingness to conduct empirical research projects in the future		
Yes	140	70.0
No	60	30.0

^a^Variable computed based on two questions related to qualitative and quantitative methods being part of education

^b^Data missing for two cases for ‘proportion of time spent on empirical work’ and one case for ‘proportion of time spent on normative work’. ^c^these were not continuous variables and thus mean and range cannot be presented

### Who is doing empirical research in bioethics?

Only 25 (12.5%) participants have never collected, analysed, or supervised empirical research projects and are not currently planning an empirical study. Of these ‘non-users of empirical research’, 20 considered it possible that they would apply empirical methods for future studies whereas five did not. Four of them provided additional information on their reasons for never engaging in empirical work: the conviction that empirical work does not answer their research questions (*n* = 2) or that their work was philosophical in nature and requires no empirical methods (*n* = 1), and ‘no interest in empirical bioethics’ (*n* = 1).

Most of the respondents (*n* = 175; 87.5%) indicated that they have used or use empirical methods, that is, they have at some point collected, and/or analysed, and/or supervised empirical research, and/or are currently working on an empirical project. They comprise our ‘empirical researchers’ group. In the following three parts of the results section, only this group will be included in the analyses. A total of 170 out of all 175 (97.1%) empirical researchers were also currently working on (at least) an empirical research project.

### Methodological characteristics of the ‘empirical researchers’

Of the ‘empirical researchers’ (*N* = 175), 65.1% (*n* = 114) have received training in qualitative methods, and 62.3% (*n* = 109) have received training in quantitative methods as part of their education. More than a fifth (*n* = 40; 22.9%) of the ‘empirical researchers’ never received qualitative or quantitative research training. These ‘empirical researchers’ who had no prior methodological training learned to use empirical methods by working on research projects (*n* = 29), reading about methodology (*n* = 26), or reading exemplary papers about studies using similar methods as the ones they wished to use (*n* = 21). A third of the ‘empirical researchers’ (*n* = 60; 34.3%) had three years or less of experience with empirical research, 29.7% (*n* = 52) four to seven years of empirical research experience, and the remaining (*n* = 63, 36.0%) eight or more years of empirical research experience.

### Current and past research projects: Collaborations with other disciplines and methods used

Since it is very likely that respondents may have been working on more than one project both in the past and in the present, they were asked to respond to the question concerning a current project by keeping one project in mind, either the most recent or one on which they devoted most of their time. Almost all participants who used empirical research methods collaborated with other disciplines (see Table 3) for their past projects and planned to do so for their current projects. The three disciplines most often collaborated with were philosophy, medicine, and sociology. Collaboration most often occurred with medicine for both the past projects (*n* = 132; 75.4%) and current projects (*n* = 111; 63.4%). Philosophy was the second discipline with which respondents reported most collaborations for both past and current project (*n* = 124, 70.9%; *n* = 107; 61.1%), respectively. Only four respondents were not collaborating with any other discipline for their current project.

**Table 3:** Collaboration with other disciplines and empirical methods used

	Research so far (*N* = 175)^a^		Current research project (*N* = 170)^a^	
Collaboration with other disciplines	*N*	%	*n*	%
Medicine	132	75.4	111	63.4
Philosophy	124	70.9	107	61.1
Sociology	91	52.0	67	38.3
Psychology	61	34.1	47	26.9
Other	47	26.9	39	22.3
Anthropology	37	21.1	22	12.6
None	0	0.0	4	2.3
Methods used	n	%	n	%
Qualitative	58	33.1	87	51.2
Quantitative	13	7.4	24	14.1
Both qualitative and quantitative	104	59.4	59	34.7

^a^Number and percentage of respondents who stated “Yes”

For their past project, 59.4% (*n* = 104) revealed that they have used both qualitative and quantitative methods (Table 3). A little over half of the respondents (*n* = 87; 51.2%) stated that they are using qualitative methods for their current projects. In the current projects, 34.7% (*n* = 59) of the participants noted that both qualitative and quantitative methods are being used. Quantitative methods were used least often in the past as well as current projects.

The thematic domains of the respondents’ current empirical research projects were as follows: medical ethics (*n* = 29, 16.8%), research ethics (*n* = 24, 13.9%), end-of-life and ethics of emerging technologies (*n* = 21; 12.1% each), and clinical ethics (*n* = 19, 11%). Other domains representing less than 10% of ‘empirical researchers’ responses were mainly neuroethics, care ethics, law and ethics, public health ethics, animal ethics, and business ethics.

Almost a third of the ‘empirical researchers’ (*n* = 51, 29.1%) experienced a need to alter or adjust (at least one of) the chosen empirical methods to make it applicable for their bioethics research question. After adjusting their chosen methods, most of the total group of respondents (*n* = 40, 81.6%) considered the altered method (very) appropriate for their research question.

### Qualitative and quantitative methodological competences

Of the wide variety of possible modes of data collection in qualitative research, 137 scholars who conducted empirical research (78.3%) reported using one-on-one interviews; 84 respondents (48%) used focus group discussions; and 43 participants (24.6%) utilized participant observations. Furthermore, half (87; 49.7%) of the scholars who carried out empirical research used data from open-ended questions in questionnaires which means they work on qualitative data collected within quantitatively oriented studies. Only 13 ‘empirical researchers’ reported other modes of qualitative data collection, which included using already available qualitative or quantitative data, data from medical records, experiments/intervention studies, literature reviews, participatory methods, and workshops.

The most frequently used qualitative approach was content analysis (*n* = 101; 57.7%), followed by thematic analysis (*n* = 97; 55.4%), and grounded theory (*n* = 72; 41.1%) (Table 4). We asked the ‘empirical researchers’ to give an estimation of their level of expertise in relation to the specific approaches they said they have used (Table 5). For all the qualitative approaches listed, 6% or less considered themselves experts. Content analysis and thematic analysis were the two analytical methods with which most respondents (close to 60%) were at least familiar with.

**Table 4:** Type of methods used by empirical researchers

Study sample (*N* = 175)		
	*n* ^a^	%
Qualitative methods		
Content analysis	101	57.7
Thematic analysis	97	55.4
Grounded theory	72	41.1
Phenomenology	33	18.9
Narrative analysis	31	17.7
Discourse analysis	29	16.6
Quantitative methods		
Descriptive analysis	104	59.4
Inferential statistics	66	37.7

^a^Number of respondents stating “Yes”; Percentages listed here do not add up since participants could choose more than one qualitative approach and quantitative methods

**Table 5:** Qualitative and quantitative methodological competence (*N* = 175)

	Expert		Familiar		Beginner		Never used		Don’t know	
	*n*	%	*N*	%	*n*	%	*n*	%	*n*	%
Qualitative methods										
Thematic Analysis (*n* = 144)	6	4.2	84	58.3	22	15.3	19	13.2	13	9.0
Content Analysis (*n* = 137)	8	5.8	91	66.4	14	10.2	17	12.4	7	50.1
Grounded Theory (n = 130)	7	5.4	64	49.2	19	14.6	28	21.5	12	9.2
Phenomenology (*n* = 118)	3	2.5	29	24.6	18	15.3	51	43.2	17	14.4
Narrative Analysis (*n* = 116)	4	3.4	41	35.3	13	11.2	48	41.4	10	8.6
Discourse Analysis (*n* = 115)	4	3.5	35	33.0	15	13.0	47	40.9	14	12.2
Quantitative methods										
Descriptive statistics (*n* = 111)	6	5.4	72	73.8	18	16.2	3	2.7	2	1.8
Inferential statistics (*n* = 98)	2	2.0	53	54.1	25	25.5	5	5.1	13	13.3

With regard to the quantitative methods used, 98 ‘empirical researchers’ (56.0%) reported using surveys or questionnaires as a mode of data collection, followed by retrospective data collection from, for example, medical records (*n* = 46; 26.3%). Only six participants stated other sources of data collection such as neurophysiological methods, tests, vignettes, and experiments. More than half (*n* = 104; 59.4%) of the respondents noted performing descriptive statistics and 66 (33.7%) analysed their data through commonly used inferential statistics (e.g. t-test, Chi^2^, regression; Table 4). Most scholars who used quantitative methods (73.8%) were at least familiar with descriptive statistics (Table 5).

### Integration of the empirical and the normative

Nearly three quarters of the ‘empirical researchers’ (76%; *n* = 130) revealed that their current project entailed a normative question (Table 6). A little over one third (35.4%; *n* = 62) reported that they attempted to integrate their empirical data with a normative analysis in their completed empirical projects. Interestingly, for their current projects, nearly half as many scholars (59.8%; *n* = 101) planned to carry out normative-empirical integration.

**Table 6:** Integration of the normative and the empirical (*N* = 175)

	*n*	%
The current project entails a normative question^a^		
Yes	130	76.0
No	41	24.0
Ever carried out an integration of empirical research findings and normative analysis		
Yes	62	35.4
No	113	64.6
Planning integration of the normative and empirical for the current project^a^		
Yes	101	59.8
No	68	40.2

^a^variables have missing values

Several of the scholars (*n* = 47, 26.8%) who have carried out this integration provided more details (including references to methodological papers) about the methods that they used (a few participants referred to more than one method). The methods most often mentioned were ‘(wide) reflective equilibrium’, or ‘reflective balancing’ (*n* = 13; sources provided [4–6, 20, 21]), and ‘dialogical empirical ethics’ (*n* = 6; [22, 24]). Other methods mentioned were 'grounded theory' (*n* = 3), ‘integrative empirical ethics’ (*n* = 2 [7, 25]), ‘grounded moral analysis’ (*n* = 2; [26]), ‘hermeneutical approach to bioethics’ (*n* = 1; [27]), ‘real world ethics approach’ (*n* = 1; [28]), ‘empirical ethics and contextualism’ (*n* = 1; [29]), ‘critical realism’ (*n* = 1; [30]), and ‘symbiotic ethics’ (*n* = 1; [31]). Interestingly, other methods reported for carrying out the integration of normative and empirical were methods that have not been designed for such purpose: ‘interpretive phenomenology’ (including IPA (*n* = 3)), ‘thematic analysis’ (*n* = 2), ‘mixed methods’ (*n* = 2), ‘systematic review method’ (*n* = 2; [32–34]), ‘content analysis’ (*n* = 1), ‘virtue ethics approach’ (*n* = 1), ‘naturalistic inquiry’ (*n* = 1), ‘focus group analysis’ (*n* = 1), ‘action research’ (*n* = 1); ‘hermeneutics’ (*n* = 1), ‘naturalistic inquiry’ (*n* = 1), ‘philosophical analysis’ (*n* = 1), and ‘participatory approach’ (*n* = 1). Three participants also noted that they did not use a specific method when integrating the empirical and the normative.

### Attitudes towards the use of empirical research methods

The attitudes of all respondents (*N* = 200) were studied using eight Likert-type questions. Almost all respondents agreed to the following two statements on the value of empirical research in bioethics: “I find it positive that empirical research is done in the field of bioethics” (*n* = 189, 94.5%); and “Empirical research is valuable in describing the context of an ethical problem” (*n* = 193, 96.5%). For the two questions concerning normative and empirical integration, 156 respondents (78.0%) thought that “empirical research is valuable for normative analysis” whereas only 57 respondents (28.5%) felt that “there is/are clear method(s) to integrate empirical findings into normative analysis.” Towards the noted ‘empirical trend in bioethics,’ a quarter of the sample (*n* = 48, 24.0%) agreed that “the trend towards empirical research in bioethics is leading bioethics away from normative work.” Finally, regarding research methodology and competences, 85 (42.5%) respondents agreed that “bioethics needs its own empirical research methodology.” The majority, 186 (93.0%) of respondents, agreed that “researchers in the field of bioethics should have the skills to interpret empirical findings,” while over half (110, 55.0%) believed that “researchers in the field of bioethics should have the skills to conduct their own empirical research.” In Table 7 additional information are presented for our sample of ‘empirical researchers’ and responses are differentiated by those who have at least once attempted to integrate the normative and the empirical versus those who did not.

**Table 7:** Attitudes of bioethics scholars towards the use of empirical research^a^

	All respondents (*N* = 200)		All empirical researchers (*N* = 175)		Integrators of Normative and Empirical (*N* = 62)^b^		Non-Integrators of Normative and Empirical (*N* = 113)	
	*n*	%	*N*	%	*n*	%	*n*	%
I find it positive that empirical research is done in the field of bioethics^c^	189	94.5	170	97.1	62	100	108	95.6
Empirical research is valuable in describing the context of an ethical problem	193	96.5	169	96.6	61	98.4	108	95.6
Empirical research is valuable for normative analysis^c^	156	78.0	140	80.0	56	90.3	84	74.3*
There is/are clear method(s) to integrate empirical findings into normative analysis	57	28.5	50	28.6	16	25.8	34	30.1
I fear that the trend towards empirical research in bioethics is leading bioethics away from normative work	48	24.0	39	22.3	11	17.7	28	24.8
Bioethics needs its own empirical research Methodology^c^	85	42.5	76	43.4	23	37.1	53	46.9
Researchers in the field of bioethics should have the skills to interpret empirical findings	186	93.0	162	92.6	56	90.3	106	93.8
Researchers in the field of bioethics should have the skills to conduct their own empirical research^c^	110	55.0	100	57.1	30	48.4	70	61.9

^a^Respondents answering ‘strongly agree’ or ‘agree’ within each category

^b^‘Integrators of normative and empirical’ was defined as respondents who answered positive to the question: “Have you ever carried out a study to integrate empirical research findings and normative analysis”

*p ≤ 0.05. *p*-value based on Chi^2^ test were run for selected questions (^c^) based on descriptive responses. We compared ‘integrators’ and ‘non-integrators’ of empirical research methods. Bonferroni correction applied for multiple testing

Based on a combination of two questions (the question about having integrated the empirical with the normative in the past and the question about plans for future project), a variety of profiles of scholars were found (see Additional file 3). That is, over two thirds of the 62 respondents who attempted to integrate in the past (*n* = 43; 69.4%) also intended to do so in future projects. Interestingly, that also means that 30.6% (*n* = 19) of them would not do so (anymore or just yet). One third (*n* = 58; 33.1%) of the ‘empirical researchers’ did not do so in the past but intended to integrate the empirical and the normative in future projects. In this survey, we could not go further into the reasons for not wanting to integrate in future projects. Additional file 3 further presents differences in attitudes towards the use of empirical research for these different profiles of scholars.

## Discussion

This survey presents a descriptive picture of the empirical research conducted by scholars in 12 European countries as well as the methods they used, their methodological training, and their attitudes towards empirical research in bioethics. Being the first of its kind to explore this topic from the perspectives of scholars in the field, ranging from doctoral students to seasoned professors, this study provides important data depicting the current state of empirical research in bioethics in the sampled countries.

In our study, most of the participants (85%) were identified as ‘empirical researchers’ because they collected, analyzed or supervised empirical research, or were preparing to conduct empirical research in the future. The vast majority of the ‘empirical researchers’ (96.6%) were positive about the value of empirical research in bioethics in describing the context of an ethical problem and most of them (80.0%) felt that empirical research is valuable for normative analysis. More than a fifth of these ‘empirical researchers’ never received formal qualitative or quantitative research training and only a very small proportion of the ‘empirical researchers’ classified themselves as an expert in the qualitative approach or the quantitative methods that they used. The researchers without methodological training said they learned methodological skills by working on the research projects, reading about methodology, or reading papers where methods were used that they intended to implement in their projects. These findings might reflect the field’s relatively recent increase in the use of empirical research [35], meaning that bioethics scholars who are not trained in empirical methods may have to adapt to this new reality and learn the methods along the way.

The responses of the ‘empirical researchers’ to the attitude questions revealed that almost all (92.6%) of them agreed that researchers in the field should have skills to interpret empirical findings, whereas 57.1% agreed that researchers in the field should have the skills to conduct empirical research. These findings may reflect the respondents’ general view of the profile of an empirical researcher in bioethics, that is, a scholar who – apart from having bioethics training - has some training in the use of methods but is not completely oriented to empirical research.

We should be careful about drawing conclusions from the first results presented in Additional file 3 concerning attitudes of the different profiles of scholars towards empirical research in bioethics. That is, the differences in attitudes found between the different profiles of scholars according to their past integration attempts and their integration plans are difficult to interpret and show mainly the need to continue exploring this in more depth.

There are some interesting differences in the methods reported by the ‘empirical researchers’ in our study compared to those reported in the systematic review of Borry et al. [2] of nine bioethics journals between 1990 and 2003. In our study, mostly a combination of both qualitative and quantitative methods was used, followed by qualitative methods, whereas the methods most used in the review of Borry et al. [2] tended to be based on one method. There, most (64.6%) of the manuscripts published made use of quantitative methods, followed by qualitative methods (32.2%), and a mix of both methods in only 3.2% of the published works [2]. These differences are, apart from other factors such as the time of the studies, partly due to differences in the methods used and questions posed in the two studies. That is, whereas the systematic review was based on publications, in our study, we asked bioethics scholars about the methods they used for their projects. A project is likely to result in more than one publication and a published paper tends to reflect one specific aim of a project and thus depicts only the corresponding method used to address the specific aim. Also, mixed-methods studies are often difficult to publish because of the word limits imposed by journals. In sum, there are many possible factors that may explain the differences in methods reported in both studies. However, it still is interesting to look at our data against the methods used in published papers. We also found that the data collection methods utilized by the ‘empirical researchers’ were similar to what is reported in available literature [1, 2]. The most used qualitative approaches by respondents of our study (content analysis, thematic analysis, and grounded theory) were also the ones the ‘empirical researchers’ felt most competent about. With regard to quantitative analysis, most respondents were also competent with descriptive and inferential statistics, but very few saw themselves as experts.

As an interdisciplinary field, bioethics uses methodology originating from disciplines such as sociology, psychology, and anthropology [36]. These were also the disciplines where our ‘empirical researchers’ collaborated with (in addition to philosophy and medicine). In the process of borrowing methods from other disciplines, approximately one-third of the ‘empirical researchers’ felt the need to adjust their chosen empirical methods but also felt they succeeded in doing so. Almost half (43.4%) of the scholars engaged in empirical research thought that bioethics needs its own methodology. Whether bioethics needs its own specific methodology is an open issue [19]. However, this leads to several important questions that should be explored in future research: What are the needs related to empirical research in bioethics that these researchers believe cannot be addressed without a customized method? What are the shortcomings of the methods that we currently use from other disciplines to justify such a customized method for bioethics? In what way would these “new” methods be considerably different from other disciplines?

Most of the ‘empirical researchers’ (80%) felt that empirical research in bioethics has value for normative analysis, further explaining why integration of the normative and the empirical seemed like a valuable endeavour [28, 37]. However, more ‘empirical researchers’ intended to carry out normative-empirical integration (59.8%) than those who had done so (35.4%). This discrepancy is highly interesting but cannot be explained by our data. Nevertheless, at least the intentions of the scholars are in line with the growing body of work that emphasizes the role of empirical data for a normative analysis [8–10, 12, 38] and the increase in publications about new methods of how this coming together of empirical and the normative could be achieved [5–7, 17, 22, 23, 28]. At the same time, the discrepancy may also reflect the general lack of agreement about when and how to carry out this integration [11, 19]. This is in line with the finding that most of our ‘empirical researchers’ (71.4%) disagreed with the statement that there was a clear way to integrate the normative and the empirical. The lack of clarity as to when and how to carry out the integration is evident from the many methods that the ‘empirical researchers’ reported using [4, 6, 20, 24, 26, 31], especially the ‘unusual methods’ that they reported (such as thematic analysis, content analysis, and focus groups). The latter finding shows the limit of self-reports in that we cannot question the scholars any further. This finding indicates that scholars have different notions of what counts as integration. Finally, the difference between the proportion of scholars that have integrated empirical data in normative analysis and the proportion of scholars planning to do so may be merely reflecting the willingness of these scholars to integrate the normative and the empirical when planning their project, while in the end, this objective is not achieved for many of them. Our findings, however, also signpost a possible overestimation of the proportion of ‘empirical researchers’ in bioethics who have integrated the empirical and the normative because it is questionable that the methods they say they have used, can in fact be used for that purpose. In answer to this, it will be important for the academic community to further debate this topic and offer at least some guidance to scholars as to what are the thresholds of what counts as integration. In addition, given our current search for methods and the present diversity in proposals of integrative methodology, we need to ask scholars to be at least transparent about the methods they have used.

An intriguing finding is that nearly a quarter of the empirical scholars who attempted to integrate the empirical and the normative, were not planning to do so in future projects. This could be related to the nature of the future research projects as well as to factors relating to the practice of integrating. For example, they may be more convinced that empirical research is valuable for normative analysis while at the same time decide not do this themselves because they have a more clear view of the difficulties one may encounter and do not consider themselves up to the job (anymore or just yet) or there is lack of research funding to take this step of integrating the normative and the empirical. These are however speculations and not explanations of the findings, which underline the need to examine this in more detail. In future research, it would be interesting to find out how scholars who made such attempts look back on their endeavours and why they would or would not intend to integrate the empirical and the normative in their future work.

### Limitations

First, the findings of the study are limited to 12 European countries therefore precluding more generalising conclusions. A second and a more important limitation of the study is the problem of representativeness of the studied sample. Even for the studied countries, we cannot assume that all researchers working in the field of bioethics were reached through our procedure since we sampled specifically those who were affiliated with a bioethics centers. Although we collected information through several channels, it is possible that we have missed some bioethics centers. Third, the response rate could be considered reasonable in comparison to other social science studies. Nevertheless, the fact that many identified bioethics scholars did not respond to the study also means that the voices of more than half of the sample are not known. It is likely that this has led to a selection bias, namely that many of the non-respondents were more normatively oriented bioethics scholars who simply considered the topic of the survey as not being relevant for them. Thus our estimation of the proportion of bioethics scholars who conduct empirical research (or plan to do so) is an overestimation. This is particularly unfortunate since we aimed to gather data also from researchers who did not conduct empirical research on questions unrelated to methodology. However, this study is valuable in that it provides insight into for instance, bioethics scholars who use empirical research methods, their background, the methods they use as well as their level of confidence with the most common methods and their attempts (and the methods for that) to integrate empirical findings in normative analysis. Furthermore, the study is novel in that it gathered data from the perspectives of bioethics scholars and in that way, it is a much needed addition to the studies based on published papers from which methodological information was extracted [1, 2]. The added value of surveying bioethics scholars is shown for example in findings such as the discrepancy between the proportion of ‘empirical researchers’ who say they have integrated the empirical with the normative and those who planned to do so for their current work. Therefore, this survey is an important first step in mapping the empirical work done in the field so that it is possible to have a well-informed debate about how things should be done.

## Conclusions

The empirical research of most of the ‘empirical researchers’ in this study remained descriptively oriented. Many did not integrate the empirical and the normative. Although it is not a pre-requisite that all empirical research in bioethics should seek to include normative analysis, scholars have noted the importance of such empirical work in bioethics when it incorporates good quality normative analysis [28, 37]. The fact that ‘empirical researchers’ mentioned a multitude of methods for integrating the normative and the empirical, and that methods were mentioned that - as such – are in no way designed for that purpose or accepted as suitable methods shows that scholars are in search of methods. Hence, there is a need of clear guidance as to the possible methods and how a particular method could be selected and used. Such guidance could provide an answer to the discrepancy that we found between those who intend to carry out empirical and normative integration and those who have actually done so. In general, the studied bioethics scholars clearly thought that empirical research in bioethics is valuable for the field. Thus, let us move the field forward by trying to agree on methods and training programs not only related to the empirical methods needed to design, collect, and analyse empirical data but also to learn how this integration of the empirical and the normative can be done. There is a need to evaluate the current educational programs in bioethics and to implement training in empirical research methods that will at least enable bioethics scholars to read and critically assess empirical research in bioethics. Apart from that, there is need for training that will allow those scholars to also design and conduct empirical research, as well as use the results of such studies in relation to a normative analysis. These trainings must also underline the necessity of upholding the methodological standards of both bioethics and the disciplines that provide the social science methods as noted by many other scholars [16–18, 39]. Apart from that, and in absence of a state of the art of the use of empirical research in bioethics, transparency about the methods being used is of utmost importance.

## Additional files

Additional file 1: Figure S1. Selection of European countries included in the study. This figure provides details on how the 12 countries were selected for this study. (DOCX 36 kb)

Additional file 2 The Use of Empirical Research in Bioethics: Questionnaire. The is the study questionnaire, which our participants received and includes the survey logic. (DOCX 27 kb)

Additional file 3: Table S8. Attitudes of bioethics scholars towards the use of empirical research according to their past behavior and their plans for the future. This table provides further data on attitudes towards the use of empirical research for our study sample based on profiles of scholars that we created using their responses to two different questions. (DOCX 24 kb)

## Abbreviations

EACME: European Association of Centres of Medical Ethics
ESPMH: European Society for Philosophy of Medicine and Healthcare

## Footnotes

1 The term ‘bioethics institutes’ will be used to refer to all institutes or centers of bioethics, ethics, biomedical ethics, history of medicine and biomedical ethics, and applied ethics.
