# Supplementary material for: The use of empirical research in bioethics: a survey of researchers in twelve European countries
Source: BMC Med Ethics. 2017 Dec 22;18:79. doi: 10.1186/s12910-017-0239-0 (PMC5741864; doi:10.1186/s12910-017-0239-0)
Supplement: Supplementary file 1 — Selection of European countries included in the study. This figure provides details on how the 12 countries were selected for this study. (DOCX 36 kb) [file 12910_2017_239_MOESM1_ESM.docx]

**Additional file 1**

Figure 1: Selection of European countries included in the study

Additional 26 institutes from **12 countries** were added using EACHME and ESPHM

38 bioethics centers identified using personal network from **13 countries**

**12 countries included** in the study:

Belgium, Denmark, Germany, Ireland, Moldova, Netherlands, Norway, Romania, Switzerland, Spain, Sweden, and United Kingdom

Excluded 8 countries*: Iceland, Italy, Portugal, France, Czech Republic, Bulgaria, Slovakia, and Russia

64 bioethics centers identified **from 20 countries** (from the above two approaches)

Excluded 8 countries*: Iceland, Italy, Portugal, France, Czech Republic, Bulgaria, Slovakia, and Russia

35 bioethics centers were included from the 12 countries

469 bioethics scholars affiliated with the 35 centers formed the study population

* Countries for which no contact information of members could be obtained in more than half of the centers were excluded.
