# Supplementary material for: The use of empirical research in bioethics: a survey of researchers in twelve European countries
Source: BMC Med Ethics. 2017 Dec 22;18:79. doi: 10.1186/s12910-017-0239-0 (PMC5741864; doi:10.1186/s12910-017-0239-0)
Supplement: Supplementary file 2 — The Use of Empirical Research in Bioethics: Questionnaire. The is the study questionnaire, which our participants received and includes the survey logic. (DOCX 27 kb) [file 12910_2017_239_MOESM2_ESM.docx]

**Additional file 2:**

**The Use of Empirical Research in Bioethics: Questionnaire**

Q1 The Use of Empirical Research in Bioethics The following questions concern your views and attitudes towards empirical research in bioethics:

Q2 I find it positive that empirical research is done in the field of bioethics.

- Strongly agree
- Agree
- Neither agree nor disagree
- Disagree
- Strongly disagree

Q3 Empirical research is valuable in describing the context of an ethical problem.

- Strongly agree
- Agree
- Neither agree nor disagree
- Disagree
- Strongly disagree

Q4  Empirical research is valuable for normative analysis.

- Strongly agree
- Agree
- Neither agree nor disagree
- Disagree
- Strongly disagree

Q5  There is/are clear method(s) to integrate empirical findings into normative analysis.

- Strongly agree
- Agree
- Neither agree nor disagree
- Disagree
- Strongly disagree

Q6 I fear that the trend towards empirical research in bioethics is leading bioethics away from normative work.

- Strongly agree
- Agree
- Neither agree nor disagree
- Disagree
- Strongly disagree

Q7 Bioethics needs its own empirical research methodology.

- Strongly agree
- Agree
- Neither agree nor disagree
- Disagree
- Strongly disagree

Q8 Researchers in the field of bioethics should have the skills to interpret empirical findings.

- Strongly agree
- Agree
- Neither agree nor disagree
- Disagree
- Strongly disagree

Q9 Researchers in the field of bioethics should have the skills to conduct their own empirical research.

- Strongly agree
- Agree
- Neither agree nor disagree
- Disagree
- Strongly disagree

The following questions concern your experience with (and reasons for) using or not using empirical research in the field of bioethics:

Q10 Have you ever collected empirical data (e.g., surveys, interviews, focus groups) for your research in the field of bioethics?

- Yes
- No

Q11 Have you ever analysed empirical data (using either qualitative and/or quantitative analytic techniques) for your research in the field of bioethics?

- Yes
- No

Q12 Have you ever supervised the collection and/or analysis of empirical research for your research in this field of bioethics?

- Yes
- No

Q13 Are you currently preparing an empirical research project (i.e., wrote an empirical research proposal, planning to collect and/or analyse data)?

- Yes
- No

Display This Question:

If Have you ever collected empirical data (e.g., surveys, interviews, focus groups) for your researc... No Is Selected

And Have you ever analysed empirical data (using either qualitative and/or quantitative analytic tech... No Is Selected

And Have you ever supervised the collection and/or analysis of empirical research for your research i... No Is Selected

And Are you currently preparing an empirical research project? No Is Selected

Q14 Do you see a possibility of applying empirical methods for your studies in bioethics in the future?

- Yes
- No

If Yes Is Selected, Then Skip To  Socio-demographic and profess...

Display This Question:

If Do you see a possibility of applying empirical methods for your studies in bioethics in the future? No Is Selected

Q15 Could you please describe briefly, why not?

If Could you please describe b... Is Displayed, Then Skip To  Socio-demographic and profess...

Q16 In the following, we are interested to know your General Experience with using empirical research methods:

Q17 Which of the following disciplines do you usually collaborate with? (select all that apply)

- Sociology
- Anthropology
- Psychology
- Medicine
- Philosophy
- NONE
- OTHER ____________________

Q18 For your empirical research up to this moment, which methods have you been /are you using?

- ONLY Qualitative methods
- ONLY Quantitative methods
- BOTH Qualitative and Quantitative methods

Q19 Have you ever carried out a study to integrate empirical research findings and normative analysis?

- Yes
- No

Display This Question:

If Have you ever carried out a study to integrate empirical research findings and nor... Yes Is Selected

Q20 Which method(s) have you used / are you using for integration of empirical data with normative analysis? (Please give the name of the method/approach. A reference for this method will be appreciated: author(s) and year of publishing will suffice.)

Display This Question:

If For your empirical research up to this moment, which methods have you been /are you using? ONLY Quantitative methods Is Not Selected

Q21 Which qualitative data collection mode do you use/have you used (select all that apply):

- One-on-one interviews
- Focus Group Discussions
- (participant) Observation
- Data from open-ended questions in questionnaires
- OTHER ____________________

Display This Question:

If For your empirical research, which methods have you been /are you using? Only Quantitative methods Is Not Selected

Q22 Please indicate which qualitative approaches you use/have used (select all that apply):

- Phenomenology
- Thematic Analysis
- Content Analysis
- Discourse Analysis
- Grounded Theory
- Narrative Analysis
- Other qualitative analysis 1 ____________________
- Other qualitative analysis 2 ____________________
- Other qualitative analysis 3 ____________________

Display This Question:

If For your empirical research, which methods have you been /are you using? Only Quantitative methods Is Not Selected

Q23 Please indicate your level of experience in the qualitative approaches you selected in the previous question:

|  | Expert in this method | Know this method very well | Familiar with this method | Beginner in this method | Never used this method | Don't Know this method at all |
| --- | --- | --- | --- | --- | --- | --- |
| Phenomenology |  |  |  |  |  |  |
| Thematic Analysis |  |  |  |  |  |  |
| Content Analysis |  |  |  |  |  |  |
| Discourse Analysis |  |  |  |  |  |  |
| Grounded Theory |  |  |  |  |  |  |
| Narrative Analysis |  |  |  |  |  |  |
| Other qualitative analysis_1 |  |  |  |  |  |  |
| Other qualitative analysis_2 |  |  |  |  |  |  |
| Other qualitative analysis_3 |  |  |  |  |  |  |

Display This Question:

If For your empirical research up to this moment, which methods have you been /are you using? ONLY Qualitative methods Is Not Selected

Q24 Which mode of data collection do you use: (check all that apply)

- Closed-ended questionnaire/survey
- Retrospective data collection (e.g. medical records)
- OTHER ____________________

Display This Question:

If For your empirical research, which methods have you been /are you using? ONLY Qualitative methods Is Not Selected

Q25 Which type of quantitative analysis do you generally perform: (select all that apply)

- Descriptive analysis (e.g. frequency, mean, median, cross-tabs)
- Inferential statistics (e.g. t-test, regression, ANOVA)
- OTHER ____________________

Display This Question:

If For your empirical research, which methods have you been /are you using? ONLY Qualitative methods Is Not Selected

Q26 Please indicate your level of experience with the quantitative analysis that you selected in the previous question:

|  | Expert in this method | Know this method very well | Familiar with this method | Beginner in this method | Never used this method | Don't Know this method at all |
| --- | --- | --- | --- | --- | --- | --- |
| Descriptive analysis |  |  |  |  |  |  |
| Inferential statistics |  |  |  |  |  |  |
| Other (that you specified above) |  |  |  |  |  |  |

Q27 Please now think about ONE PROJECT which you are currently working on that involves empirical research. If you are currently involved in several research projects, please think about the one project that takes up most of your time. If you are currently not involved in any research, please think of the last empirical research project that you worked on.

Q28 What is the thematic domain of this project?

- End-of-life
- Research ethics
- Clinical ethics
- Medical ethics
- Law and ethics
- Reproductive ethics
- Ethics of emerging technologies
- Neuroethics
- Care ethics
- Other ____________________

Q29 Does your research entail a normative question?

- Yes
- No

Q30 Which empirical method are you using for the project?

- Qualitative
- Quantitative
- BOTH qualitative and quantitative

Display This Question:

If Which empirical method are you using for the project? Quantitative Is Not Selected

Q31 Please which qualitative approach are you using:

- Phenomenology
- Thematic Analysis
- Content Analysis
- Grounded Theory
- Narrative Analysis
- OTHER ____________________
- NONE

Q32 Was there a need to alter or adjust (at least one of) the chosen empirical methods to make it applicable for your bioethics research question?

- Yes
- No

Display This Question:

If Was there a need to alter or adjust (at least one of) the chosen empirical methods to make it app... Yes Is Selected

Q33 To what degree do you consider the chosen (adjusted) empirical methods appropriate to address your research question?

- Extremely appropriate
- Appropriate
- Neither appropriate nor inappropriate
- Inappropriate
- Extremely inappropriate

Q34 If you wish to add anything on this need to alter the empirical method, please use this space:

Q35 For your project, what have you planned?

- Empirical data collection and their analyses
- Both empirical and normative work (how or which method of integration are you planning, we would appreciate if you would provide a reference: author(s) and year of publishing will suffice) ____________________

Q36 Which of the following disciplines are involved in this project? (Select all that applies)

- Sociology
- Anthropology
- Psychology
- Medicine
- Philosophy
- OTHER ____________________
- NONE

Q37 Optional question: If you wish to explain more about what you think is required of empirical methods to be appropriate for bioethics, please use the space below:

Q38 Socio-demographic and professional background:

Q39 Education

- PhD or equivalent
- Master's degree
- Other (please elaborate) ____________________

Q40 Please specify which domain/discipline was your degree from _________________

Q41 Please indicate if you have had courses on qualitative methods classes as part of your education:

- Yes
- No

Q42 Please indicate if you have had courses on quantitative methods classes as part of your education:

- Yes
- No

Display This Question:

If Please indicate if you have had courses on qualitative methods classes as part of your education: No Is Selected

And Please indicate if you have had courses on quantitative methods classes as part of your education:  No Is Selected

Q43 If methodology was not part of your education, how did you learn empirical research methodology? (Choose all that apply)

- By reading about methodology
- By working on research projects
- By reading papers that describe examples of similar methods I wanted to use
- Through courses offered at my university
- Through other courses offered at my university (outside my curriculum)
- Through courses offered at other universities (e.g., Summer methods school)
- DOES NOT APPLY ____________________

Q44 Please select your current position:

- Full-Professor
- Associate/Assistant Professor
- Senior Researcher/Project Manager/Senior lecturer
- Post-doctoral scholar/Junior Lecturer
- PhD student/ Research assistant

Q45 Do you supervise PhD student(s)?

- Yes
- No

Q46 Years of experience with empirical research (including your PhD research work):

- Less than 1
- 1
- 2
- 3
- 4
- 5
- 6
- 7
- 8
- 9
- 10
- More than 10 years

Q47 How much of your work time (in percentage) do you dedicate to tasks related to empirical research?

- 0 - 10%
- 11 - 20%
- 21 - 30%
- 31 - 40%
- 41 - 50%
- 51 - 60%
- 61 - 70%
- 71 - 80%
- 81 - 90%
- 91 - 100%

Q48 How much of your work time (in percentage) do you dedicate to tasks related to normative ethics?

- 0 - 10%
- 11 - 20%
- 21 - 30%
- 31 - 40%
- 41 - 50%
- 51 - 60%
- 61 - 70%
- 71 - 80%
- 81 - 90%
- 91 - 100%

Q49 How many people (in percentage) in your department/institute are doing empirical research in biomedical ethics

- 0 - 10%
- 11 - 20%
- 21 - 30%
- 31 - 40%
- 41 - 50%
- 51 - 60%
- 61 - 70%
- 71 - 80%
- 81 - 90%
- 91 - 100%

Q50 Please select which age group describes your current age:

- 18 - 24
- 25 - 34
- 35 - 44
- 45 - 54
- 55 - 64
- 65 - 74
- 75 - 84
- 85 or older

Q51 Gender

- Male
- Female

Q52 Please specify your country of current (main) employment:

- Belgium
- Denmark
- Germany
- Ireland
- The Netherlands
- Norway
- United Kingdom
- Switzerland
- Spain
- Sweden
- Romania
- Moldova
- Other

Display This Question:

If Please specify your country of current (main) employment; Other Is Selected

Q53 Please specify country:
