# Supplementary material for: The use of empirical research in bioethics: a survey of researchers in twelve European countries
Source: BMC Med Ethics. 2017 Dec 22;18:79. doi: 10.1186/s12910-017-0239-0 (PMC5741864; doi:10.1186/s12910-017-0239-0)
Supplement: Supplementary file 3 — Attitudes of bioethics scholars towards the use of empirical research according to their past behavior and their plans for the future. This table provides further data on attitudes towards the use of empirical research for our study sample based on profiles of scholars that we created using their responses to two different questions. (DOCX 24 kb) [file 12910_2017_239_MOESM3_ESM.docx]

**Additional file 3**

**Table 8: Attitudes of bioethics scholars towards the use of empirical research according to their past behavior and their plans for the future**

|  | Did a study to integrate the empirical and the normative in the past^a^  (N=62) | | | | Never integrated the empirical and the normative in the past  (N=113)^a^ | | | |
| --- | --- | --- | --- | --- | --- | --- | --- | --- |
| Statements | Future plans: only empirical work^a^  (N=19) | | Future plans: Empirical and normative work^b^  (N= 43) | | Future plans: only empirical work^a^  (N=55) | | Future plans: Empirical and normative work^a^  N= 58) | |
|  |  | |  | |  | |  | |
|  | Agree^b^  n (%) | Neutral or Disagree^c^  n (%) | Agree^b^  n (%) | Neutral or Disagree^c^  n (%) | Agree^b^  n (%) | Neutral or Disagree^c^  n (%) | Agree^b^  n (%) | Neutral or Disagree^c^  n (%) |
| I find it positive that empirical research is done in the field of bioethics | 19 (100.0) | 0 (0.0) | 43 (100.0) | 0 (0.0) | 53 (96.4) | 2 (3.6) | 55 (94.8) | 3 (5.2) |
| Empirical research is valuable in describing the context of an ethical problem | 18 (94.7) | 1 (5.3) | 43 (100.0) | 0 (0.0) | 53 (96.4) | 2 (3.6) | 55 (94.8) | 3 (5.2) |
| Empirical research is  valuable for normative analysis | **18 (94.7)*** | **1 (5.3)*** | 38 (88.4) | 5 (11.6) | **38 (69.1)*** | **17 (30.9)*** | 46 (79.3) | 12 (20.7) |
| There is/are clear method(s) to integrate empirical findings into normative analysis | 5 (26.3) | 14 (73.7) | 11 (25.6) | 32 (74.4) | 15 (27.3) | 40 (72.7) | 19 (32.8) | 39 (67.2) |
| I fear that the trend towards  empirical research in bioethics is leading bioethics away from normative work | 3 (15.8) | 16 (84.2) | 8 (18.6) | 35 (81.4) | 13 (23.6) | 42 (76.4) | 15 (25.9) | 43 (74.1) |
| Bioethics needs its own empirical research Methodology | 8 (42.1) | 11 (57.9) | *15 (34.9)** | *28 (65.1)** | **20 (36.4)*** | **35 (63.6)*** | ***33 (56.9)**** | ***25 (43.1)**** |
| Researchers in the field of bioethics should have the skills to interpret empirical findings | 18 (94.7) | 1 (5.3) | 38 (88.4) | 5 (11.6) | 51 (92.7) | 4 (7.3) | 55 (94.8) | 3 (5.2) |
| Researchers in the field of bioethics should have the skills to conduct their own empirical research | **14 (73.3)*** | **5 (26.3)*** | ***16 (37.2)**** | ***27 (62.8)**** | 36 (65.5) | 19 (34.5) | *34 (58.6)** | *24 (41.4)** |

^a^ ‘The two main categories (first row of the table) was defined as respondents who answered positive to the question: “Have you ever carried out a study to integrate empirical research findings and normative analysis? The subcategories (second row of the table) was defined as respondents who answered to the question “For your future project, what have you planned?” They were categorised in two groups according to their answers: 1) ‘Empirical data collection and their analysis’ or 2) “Both empirical and normative work (after which we asked further questions about integrating both)”.

^b^ Respondents answering ‘strongly agree’ or ‘agree’ within each category.

^c^ Respondents answering ‘strongly disagree’ or ‘disagree’ or ‘neither agree nor disagree’ within each category.

*p ≤ 0.05; **p ≤ 0.01; ***p ≤0.001. p-value based on Fisher’s Exact test (Cytel Studio StatXact-8) because of the small n to compare one category of respondents with each of the other categories separately. Within each main category (‘did a study to integrate the empirical and the normative in the past’ or ‘Never integrated’), we compared those who had plans to integrate in their future work with those who did not have such plans. For all those who had future plans that ONLY included empirical work (no normative work), we compared those who integrated in the past versus those who did not do so in the past. For all those who had future plans that included both types of research (and answered further questions about the integration of both), we compared those who integrated in the past versus those who did not do so in the past.
